# Spontaneous bimanual independence during parallel tapping and sawing

**DOI:** 10.1371/journal.pone.0178188

**Published:** 2017-05-25

**Authors:** Sandra Dorothee Starke, Chris Baber

**Affiliations:** School of Engineering, College of Engineering and Physical Sciences, University of Birmingham, Edgbaston, Birmingham, United Kingdom; University of California Merced, UNITED STATES

## Abstract

The performance of complex polyrhythms—rhythms where the left and right hand move at different rates—is usually the province of highly trained individuals. However, studies in which hand movement is guided haptically show that even novices can perform polyrhythms with no or only brief training. In this study, we investigated whether novices are able to tap with one hand by matching different rates of a metronome while sawing with the other hand. This experiment was based on the assumption that saw movement is controlled consistently at a predictable rate without the need for paying primary attention to it. It would follow that consciously matching different stipulated metronome rates with the other hand would result in the spontaneous performance of polyrhythms. Six experimental conditions were randomised: single handed tapping and sawing as well as four bimanual conditions with expected ratios of 1:1 (performed with and without matching a metronome) as well as 3:4 and 4:3 (performed matching a metronome). Results showed that participants executed the saw movement at a consistent cycle duration of 0.44 [0.20] s to 0.51 [0.19] s across single and bimanual conditions, with no significant effect of the condition on the cycle duration (*p* = 0.315). Similarly, free tapping was executed at a cycle duration of 0.48 [0.22] s. In the bimanual conditions, we found that for a ratio of 4:3 (4 taps against 3 sawing cycles per measure), the observed and predicted ratio of 0.75 were not significantly different (*p* = 0.369), supporting our hypothesis of the spontaneous adoption of polyrhythms. However, for a ratio of 3:4 (3 taps against 4 sawing cycles per measure), the observed and predicted ratio differed (*p* = 0.016), with a trend towards synchronisation. Our findings show that bimanual independence when performing complex polyrhythms can in principle be achieved if the movement of one hand can be performed without paying much—if any—attention to it. In this paradigm, small rhythmic arm movements are possibly driven by an intrinsic timing which leads to spontaneous convergence on a cycle duration of around 0.5 s, while the movement of the other hand can be controlled consciously to occur at desired rates.

## Introduction

A well-known constraint on human performance is the limit to cognitive resources [1–3] and the resulting (subconscious) prioritisation amongst parallel tasks [4]. This effect becomes apparent, for example, when walking whilst in deep conversation: as the conversation becomes more demanding, people slow down or even stop walking, which may even predict the likelihood of falls in the elderly [5,6]. The coordination of multiple activities at the same time has been extensively explored in ‘dual task’ studies [1,3,7,8]. What emerges is that humans, especially without substantial training, find it inherently difficult to do two things at once, such as dissociating the movement of the left and right hand [3,9–11]. A popular example for this is the ‘tap your head and rub your tummy’ challenge, where many people struggle to execute the two different movements at the same time. The ability to dissociate movement of the left and right hand is termed ‘independence’, where research can extend to all four limbs [12] and models have been developed for sensorimotor integration [13–16]. It remains an open question which factors allow for bimanual independence, especially without prior training; to date, only a few studies report spontaneously emerging bimanual independence.

A common experimental paradigm to examine bimanual independence and sensorimotor synchronisation uses tapping studies [10,11,17], where participants are asked to tap at different rates with the left and right hand by matching auditory stimuli. These studies show that the resulting polyrhythms—rhythms where the left and right hands execute repetitive movements at different rates—are difficult to perform. This holds true especially for rates that are not simple multiples [18]; in contrast, producing identical rhythms with both hands or simple multiples such as 2:1 is reasonably easy [9,18]. The difficulty in producing polyrhythms lies specifically in the execution of two different rhythms at the same time: the component rhythms can easily be performed separately with a single hand [8,16,19,20]. In line with this affinity towards synchronisation and simple multiples, studies consistently report the tendency of humans to synchronise the movement between the two hands despite attempting to do otherwise. This emerges in ‘mirror symmetry’ [15] or movement in phase and anti-phase [21], both of which appear to be driven perceptually towards spatial symmetry, rather than through symmetrical muscle activation [22]. Tapping complex polyrhythms such as 4:3 or 5:3 is usually reserved for highly trained individuals [23,24].

Most studies on bimanual independence require participants to consciously control movement of the hands. Outside of these paradigms, it is emerging however that humans can actually move their two hands independently without prior training. A study investigating ‘haptic tracking’, where hand movement is guided by a moving target through light contact, showed that participants could move their arms at different rates spontaneously, even at the difficult 4:3 ratio [25]. Similarly, humans can perform polyrhythmic patterns with both hands after short practice (5 to 20 minutes) in tasks where movement is guided through external physical properties: this includes short episodes when describing circles of different diameters [26] or longer episodes when moving unequal levers with the target of a symmetrical output movement [27] or swinging pendulums of different weights [28]. People are also able to perform polyrhythms and other complex coordination tasks under certain experimental conditions when trying to match a complex integrated visual output in the form of a phase-space, or ‘Lissajous portrait’ [23,29,30]. These findings suggest that bimanual independence can be achieved if movement is guided through an external stimulus. Having movements follow an external guide ought to minimise the level of deliberate control required and, one would assume, lead to the movements being executed without the need to pay attention to them. Such movements would free up cognitive resources following the distinction by Neumann (1984), assuming that actions which are performed without the need to pay attention to them (sometimes classified as ‘automatic’ action) will be processed in parallel without capacity restrictions, whereas actions under conscious control are processed serially and are hence capacity limited [31].

Previously, we observed that when engaging in the simple repetitive movement task of sawing, humans spontaneously adopt a movement frequency that is highly consistent across participants and various experimental manipulations [32]. If such simple movements can be performed while paying little or no attention to them (respectively without substantial need for conscious control), one could speculate that similar to haptic tracking, complex bimanual independence should be achievable without training, since attention has to be paid to only one hand. Where we refer to ‘paying attention’, we define this as allocating the majority of conscious monitoring, or primary attention, to a given action. In the present study, we hypothesise that participants in a polyrhythmic task, where the tapping rate changes, would continue to consistently saw at the same intrinsic rate while adjusting the tapping rate to a metronome. They would hence be able to execute movement at different rates with the left and right hand and hence display spontaneous bimanual independence.

## Material and methods

### Study design

Experiments were approved under University of Birmingham ethics regulations and written informed consent was provided by all participants. Twenty-three participants (17 male, 20 right-handed) were recruited from staff and students of Birmingham University’s School of Engineering, where 13 students participated in the study for course credit.

**Fig 1:**
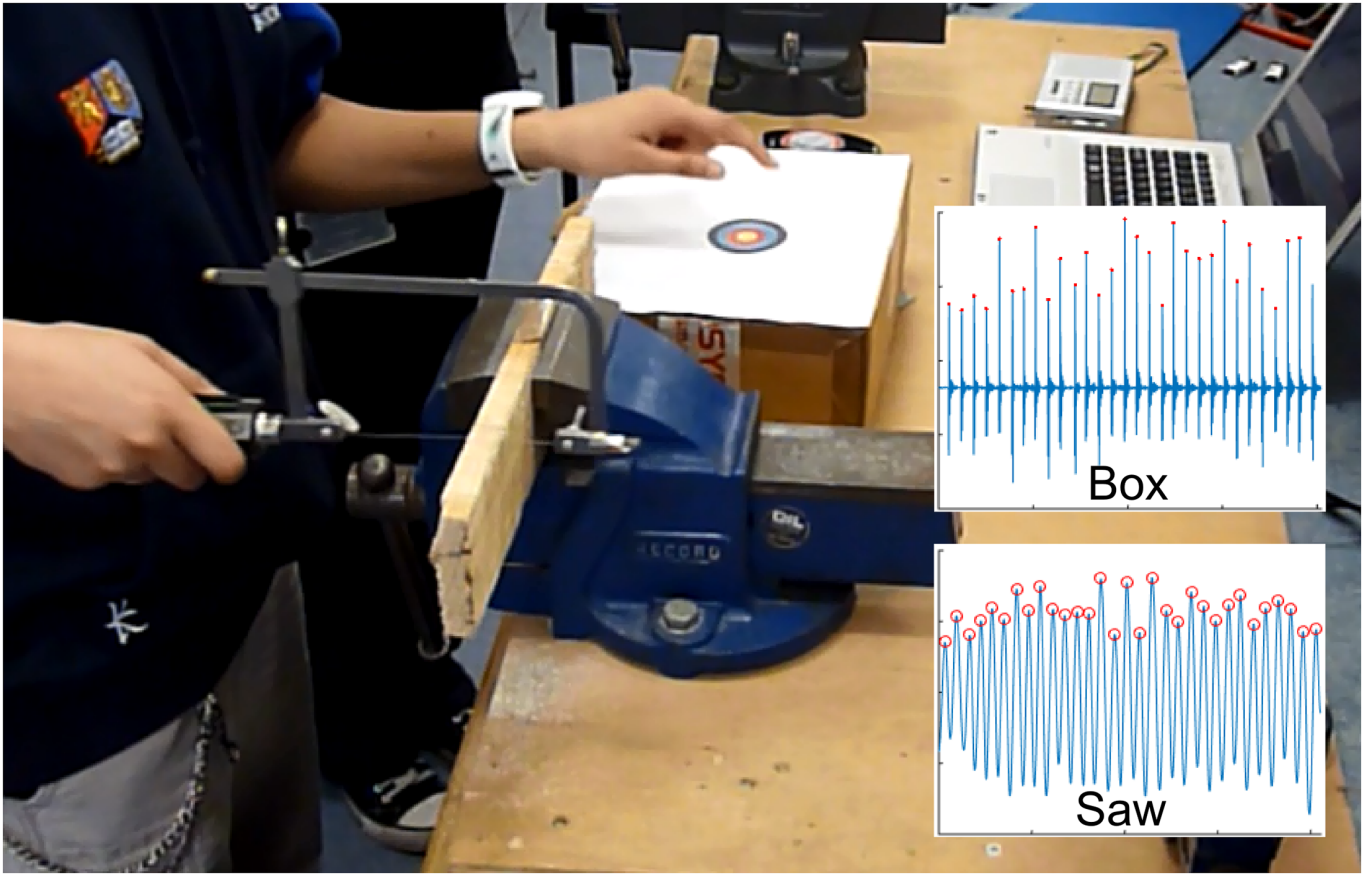
Study setup, illustrating the dual movement task where participants saw into a piece of wood while simultaneously tapping on a box to the beat of a metronome. Data from accelerometers attached to saw and box (inset) are recorded wirelessly.

Participants were asked to saw into a piece of wood with one hand and tap a box with the other hand (Fig 1), both independently and in a dual movement task. Depending on the experimental condition, tapping was performed by either matching the beat of a metronome (SQ-50V, Seiko) or tapping freely without auditory guidance. Sawing was performed freely without guidance in all experimental conditions using a piercing saw with a blade length of 8.0 cm (81 teeth per inch, tooth height 0.32 mm). The saw was custom-instrumented as described in a previous paper [33]: the handle of the saw is equipped with a tri-axial accelerometer, aligned with the orientation of the saw blade. Data were transmitted *via* Bluetooth at 120 Hz and recorded on a laptop. For the quantification of tapping cycle duration, a cardboard box was instrumented with a second tri-axial accelerometer. A circular target was attached to the top of the box and participants were asked to tap on the centre of this target. As above, data were transmitted *via* Bluetooth at 120 Hz and recorded through the same software in parallel to the sawing data stream.

The study commenced with individual hand movements, randomised across participants to be either first tapping the box or sawing into a piece of wood, and then performing the other action. Movement was timed for approximately 15 seconds in all tasks. This task duration was selected as providing sufficient data for subsequent analysis without incurring, over the course of the trials, the possibility of fatigue. The saw was always held in the preferred hand. From the first single-handed sawing trial, the preferred sawing cycle duration was calculated on-the-fly using a Fast Fourier Transformation (FFT) embedded in custom code written in Matlab (The MathWorks) while the participant filled in a questionnaire. Given our previous observation that preferred sawing rates seem to be applied consistently [32], we assumed that this would also hold true in this experiment. The preferred sawing cycle duration was hence used as the fixed rate for polyrhythmic and synchronous conditions, where the metronome beat for tapping was adjusted to result in the ratios 1:1, 4:3 and 3:4 in the bimanual tasks. Participants engaged in the following four dual movement tasks in random order:

1. Tapping and sawing synchronously at the calculated preferred sawing cycle duration (matching the metronome with tapping), respectively performing movements with a ratio of 1:1.
2. Tapping at a ratio of 4(tap):3(saw) compared to the preferred sawing cycle duration (matching the metronome with tapping; tapping expected faster than sawing).
3. Tapping at a ratio of 3(tap):4(saw) compared to the preferred sawing cycle duration (matching the metronome with tapping; tapping expected slower than sawing).
4. Tapping and sawing freely at the same time (no metronome for tapping).

The ratios of 4:3 (task 2) and 3:4 (task 3) were chosen as they are considered difficult to execute under normal circumstances [23,34] and have been used for this reason in previous research [25]. Participants were instructed to focus on the tapping task and to try to match the metronome beat as closely as possible. If participants behaved consistently with our hypothesis, the ratio between tapping and sawing cycle duration should be 0.75 for the 4(tap):3(saw) condition and 1.33 for the 3(tap):4(saw) condition. Between conditions, a brief period of auditory white noise was played using a radio set to a frequency band without a station to minimise carry-over effects.

### Data processing

Data from the instrumented saw were imported into Matlab and the raw acceleration component for the longitudinal axis of the saw blade extracted. Data from the instrumented box were imported into Matlab and the resultant raw acceleration calculated from the three acceleration components. Data were then offset corrected by subtracting the median value and double-integrated to arrive at non-calibrated displacement trajectories. A 4^th^ order, zero-lag high-pass Butterworth filter (cut-off frequency: 0.5 Hz) was used at each integration step to prevent cumulative error arising from the integration procedure. For each data stream, the range was selected that represented continuous movement. From this selection, movement cycle duration was calculated from the maxima in the signal (Fig 1). Cycle duration was calculated as the time between the identified maxima. For each participant and condition, median and interquartile range (IQR) in cycle duration were then calculated as an estimate of centrality and variation; this approach was chosen as a robust way of handling outliers arising from potential skipped beats etc. On four out of 230 occasions (participant 2, sawing and tapping in the single handed conditions; participant 3, tapping in the synchronous condition; participant 10, tapping in the single handed condition), technical problems meant that values had to be interpolated from other conditions due to lack of data. Estimates were made from data for the ‘free’ condition and either the ‘synchronous’ or ‘single hand’ condition depending on the missing data point. This procedure did not unduly skew the sample data.

For the individual hand movements, summary statistics as well as the difference and ratio between tapping and sawing were calculated from the participant-specific median values for each condition. For the four dual movement tasks, ratios of median cycle durations were calculated by dividing tapping by sawing, the set metronome beat by sawing and the set metronome beat by tapping.

### Statistics

Statistical analysis was performed in IBM SPSS Statistics 22. The significance level was set to alpha = 0.05, unless stated otherwise. Datasets for each condition were tested for normality using the Kolmogorov-Smirnov test. Since the distribution of four datasets for sawing differed significantly from a normal distribution (*p* ≤ 0.026), non-parametric statistics were performed. For the single hand task, a Wilcoxon signed-rank matched pairs test was run to examine whether the spontaneously chosen sawing and tapping cycle duration differed from each other. For the four dual movement conditions, several tests were executed. Firstly, a Friedman test was run to examine whether the experimental condition had a significant effect on the sawing and tapping cycle duration, respectively. In case of significance, post-hoc pairwise Wilcoxon tests were run with the significance level adjusted for multiple testing to alpha = 0.013 across four conditions [35]. Secondly, the sawing and tapping cycle duration was compared pairwise for each condition using Wilcoxon signed-rank matched-pairs tests. Thirdly, one-sample sign tests [36] were run to examine whether the ratio of tapping to sawing matched the expected ratios (1 for the ‘synchronous’ and ‘free’ condition, 0.75 for the ‘4(tap):3(saw)’ condition and 1.33 for the ‘3(tap):4(saw)’ condition). One-sample sign tests were run to compare the adopted tapping and sawing cycle duration to the set metronome beat for the conditions ‘synch’, ‘4(tap):3(saw)’ and ‘3(tap):4(saw)’.

To examine within-participant variation across all conditions, statistical analysis was performed following a similar strategy described for examining central tendency above. Firstly, a Friedman test was run across all five conditions for the hand performing tapping and the hand performing sawing individually. In case of significance, this was followed by post-hoc pairwise Wilcoxon tests, with the significance level adjusted to alpha = 0.010 for multiple testing across all five conditions. Secondly, to examine whether within-participant variation differed systematically between tapping and sawing for any of the dual movement conditions, pairwise Wilcoxon signed-rank matched-pairs tests were performed for each condition. Thirdly, the ratio between each participant’s variation during the single handed trial and each of the dual movement trials was calculated and a Friedman test run across all four conditions for the hand performing tapping and the hand performing sawing. In case of significance, this was followed by post-hoc pairwise Wilcoxon tests, with the significance level adjusted to alpha = 0.008 for multiple testing across all six pairs.

## Results

In the single hand task, the spontaneously adopted median [IQR] cycle duration was 0.48 [0.22] s for tapping and 0.46 [0.19] s for sawing (Table 1) and not significantly different (*p* = 0.922). Accordingly, the difference between spontaneously adopted tapping and sawing cycle duration was -0.03 [0.28] s (expected: 0 s), while the ratio was 0.95 [0.67] (expected: 1). There was no significant difference in cycle duration between the single hand task and the respective action during the dual movement task in the ‘free’ condition for either tapping or sawing (*p* ≥ 0.506).

**Table 1:** Cycle durations across all experimental conditions. This table presents the median [IQR] for the single and dual movement tasks for both, tapping and sawing, as well as the metronome beat for the three conditions in which tapping was performed to match the set beat of a metronome.

Task	Condition	Sawing cycle duration	Tapping cycle duration	Metronome
**Single**	***Saw***	0.46 [0.19]	-	-
**Single**	***Tap***	-	0.48 [0.22]	-
**Dual**	***Synchronous***	0.49 [0.26]	0.44 [0.20]	0.45 [0.20]
**Dual**	***4(tap)*:*3(saw)***	0.47 [0.23]	0.33 [0.14]	0.36 [0.20]
**Dual**	***3(tap)*:*4(saw)***	0.44 [0.20]	0.53 [0.28]	0.60 [0.26]
**Dual**	***Free***	0.51 [0.19]	0.48 [0.22]	-

**Fig 2:**
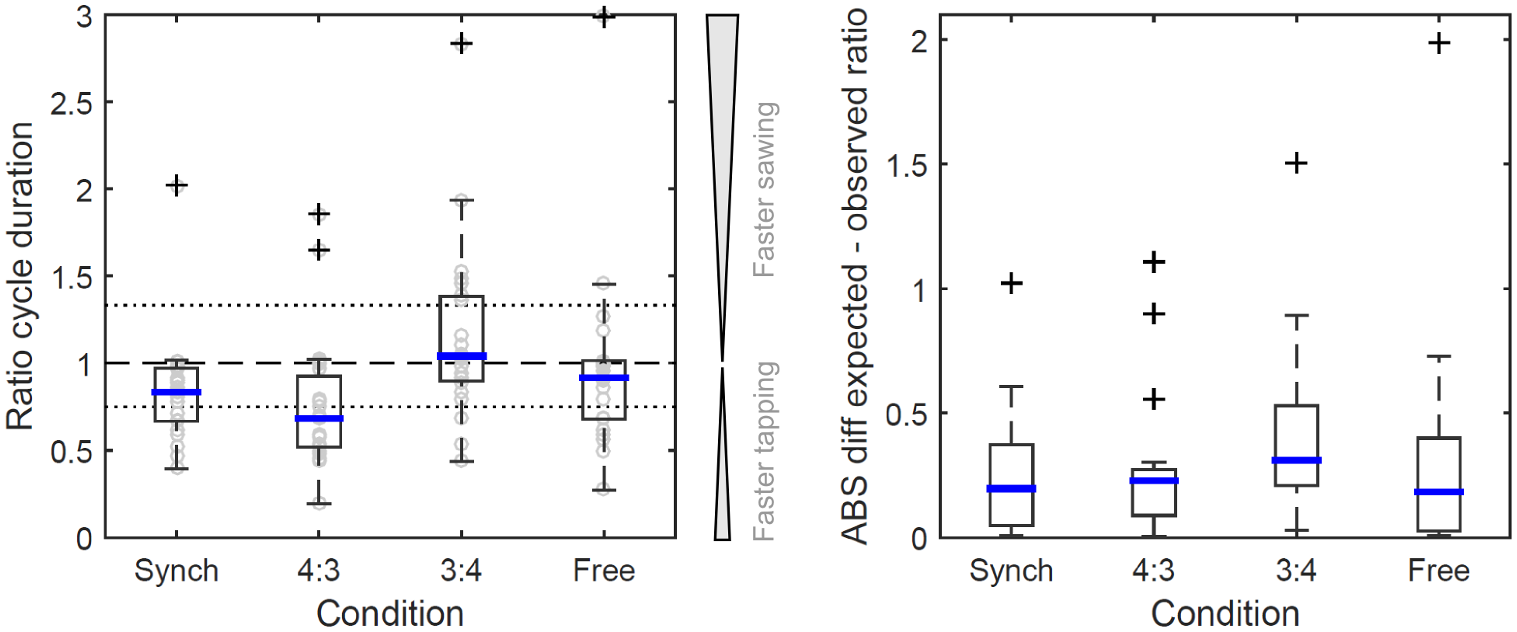
Left: boxplot for the ratios of tapping to sawing cycle duration calculated for the four dual movement tasks. Right: Absolute difference (‘ABS’) between expected and observed ratio for each participant. Experimental conditions: ‘synch’–synchronised tapping and sawing (1:1), expected since the metronome beat was set according to the preferred sawing cycle duration; ‘4:3’—4 beats tapping against 3 beats preferred sawing cycle duration per measure; ‘3:4’—3 beats tapping against 4 beats preferred sawing cycle duration per measure. Dashed line: ratio 1:1 (identical cycle duration for tapping and sawing); dotted lines: ratio 0.75 for condition ‘4:3’ (tapping duration is shorter than sawing duration) and 1.33 for condition ‘3:4’ (tapping duration is longer than sawing duration). Please note that condition labels are given as the equivalent of a frequency for ease of understanding the experimental condition.

For the dual movement tasks, median [IQR] sawing cycle duration was not significantly different between the four conditions (*p* = 0.351), ranging from 0.44 [0.20] s to 0.51 [0.19] s (Table 1). Median [IQR] tapping cycle duration, however, was significantly different between conditions (*p* ≤ 0.001), ranging from 0.33 [0.14] s to 0.53 [0.28] s. Post-hoc testing revealed significant pairwise differences between the conditions ‘4(tap):3(saw)’ compared to ‘synch’, ‘3(tap):4(saw)’ and ‘free’ (*p* ≤ 0.001) and between ‘synch’ and ‘3(tap):4(saw)’ (*p* < 0.001). Pairwise comparison between sawing and tapping cycle duration for each condition revealed a significant difference between the ‘synch’ and ‘4(tap):3(saw)’ condition (*p* ≤ 0.002), but not the other two conditions (*p* ≥ 0.085). The ratio between tapping and sawing (Fig 2) was significantly different from the expected value for the ‘synch’ and ‘3(tap):4(saw)’ conditions (*p* ≤ 0.016), but not for the ‘4(tap):3(saw)’ and ‘free’ conditions (*p* ≥ 0.075). The bias observed in the ‘synch’ condition could not explain the finding for the ‘4(tap):3(saw)’ condition, since the ratio was significantly different between these two conditions (*p* = 0.041).

**Fig 3:**
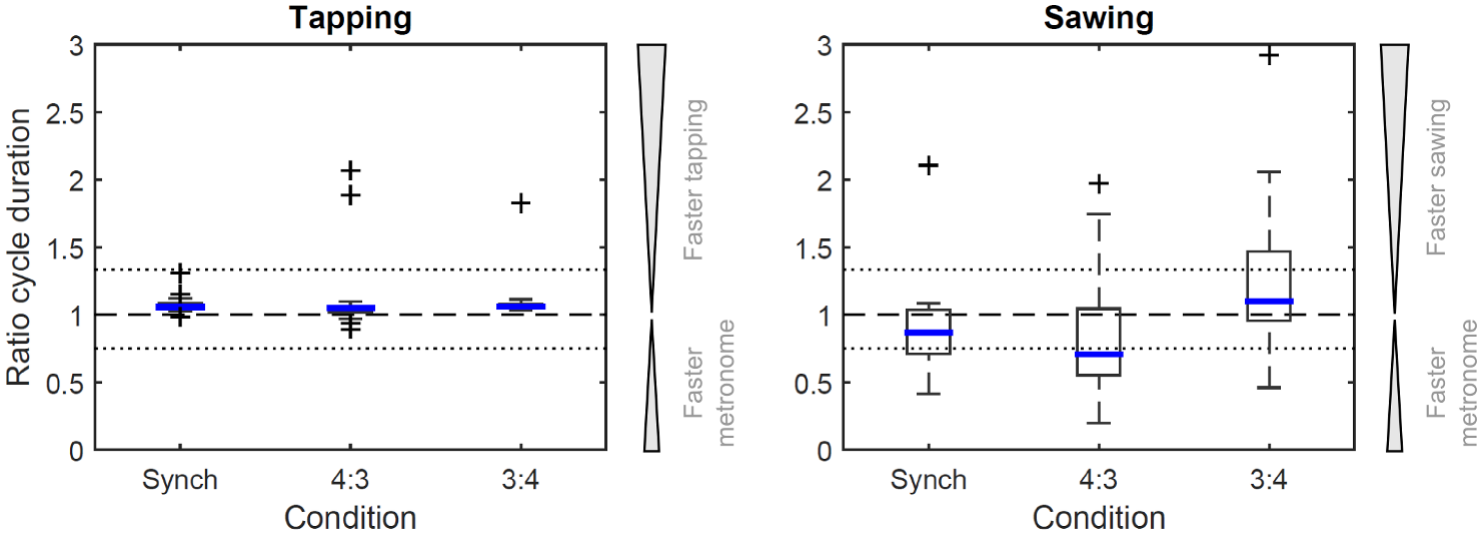
Boxplots for the ratios of the set metronome beat to tapping cycle duration (left) and sawing cycle duration (right) calculated for the three dual movement tasks where a metronome beat was matched through tapping. For experimental conditions and legend, please refer to Fig 2. The three large outliers for tapping represent participants tapping at around twice the set metronome beat, which was confirmed by video footage.

There was a very small systematic offset between tapping cycle duration and set metronome beat (Fig 3, left). This resulted in the tapping duration being significantly different from the set metronome beat for all pairwise comparisons (*p* ≤ 0.001). However, the effect was negligibly small, with a mean (SD) difference between tapping and sawing cycle duration of 0.03 (0.03) s for ‘synch’, 0.03 (0.06) s for ‘4(tap):3(saw)’ and 0.05 (0.05) s for ‘3(tap):4(saw)’. The adopted sawing cycle duration was significantly different from the set metronome beat for all conditions (*p* ≤ 0.035), where the effect was pronounced compared to results for tapping (Fig 3, right).

**Fig 4:**
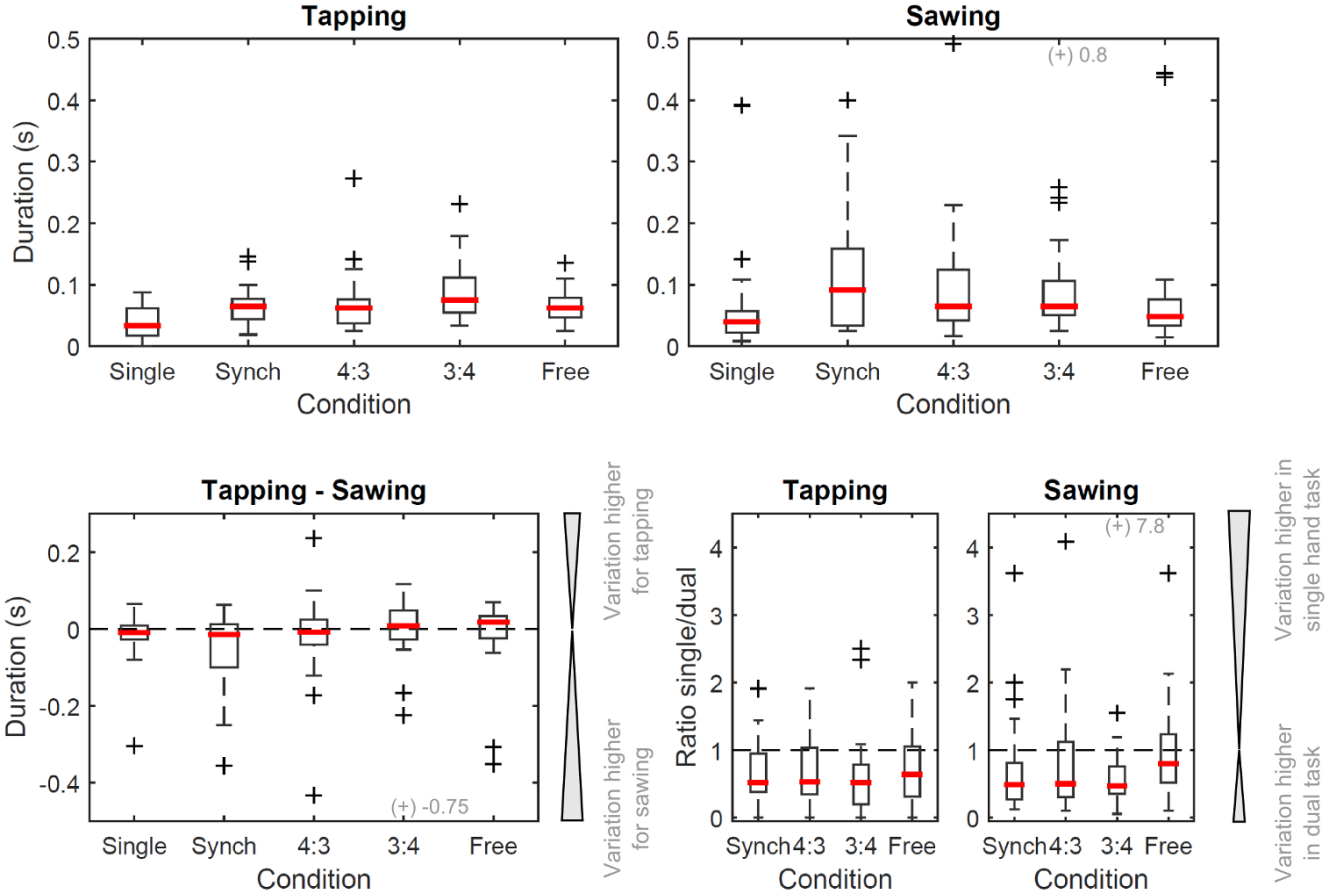
Boxplots showing within-participant variation as an index of stability. Top: within-participant IQR for the hand performing tapping (left) and the hand performing sawing (right), for both the single handed task (‘single’) and the dual movement tasks (remainder). Bottom, left: participant-specific difference between within-participant variation for the hand performing tapping and the hand performing sawing. Dashed line: no difference in variation. Bottom, right: participant-specific ratio of the variation observed during the single hand task compared to the equivalent action in the four dual movement tasks. Dashed line: no difference in variation. A value of 0.5 corresponds to half the amount of variation in the single hand task, a value of 2 corresponds to twice the variation. For further details on experimental conditions and legend, please refer to Fig 2.

Within-participant variation ranged from a median [IQR] of 0.03 [0.04] s to 0.07 [0.06] s for tapping and from 0.04 [0.03] s to 0.09 [0.13] s for sawing (Fig 4, top). The largest median within-participant variation for the hand engaging in tapping was found for the 3(tap):4(saw) condition (median [IQR]: 0.07 [0.06]), while for the hand engaging in sawing it was found for the ‘synch’ condition (0.09 [0.13]). There was a significant effect of the condition on within-participant variation for both tapping (*p* = 0.001) and sawing (*p* = 0.002). Pairwise post-hoc comparisons found a significant difference between single-handed activity and both the ‘synch’ and the ‘3(tap):4(saw)’ condition (*p* ≤ 0.004) for the hand engaging in tapping, as well as between single-handed activity and ‘3(tap):4(saw)’ (*p* = 0.002) for the hand engaging in sawing. Within-participant variation did not differ significantly between tapping and sawing for any of the dual movement conditions (*p* ≥ 0.069). The ratio between each participant’s variation in performing a given action in the single hand trial compared to its equivalent during the dual movement trials (Fig 4, bottom) was not significantly different between the four dual movement conditions for the hand performing tapping (*p* = 0.649), but for the hand performing sawing (*p* = 0.046).

**Fig 5:**
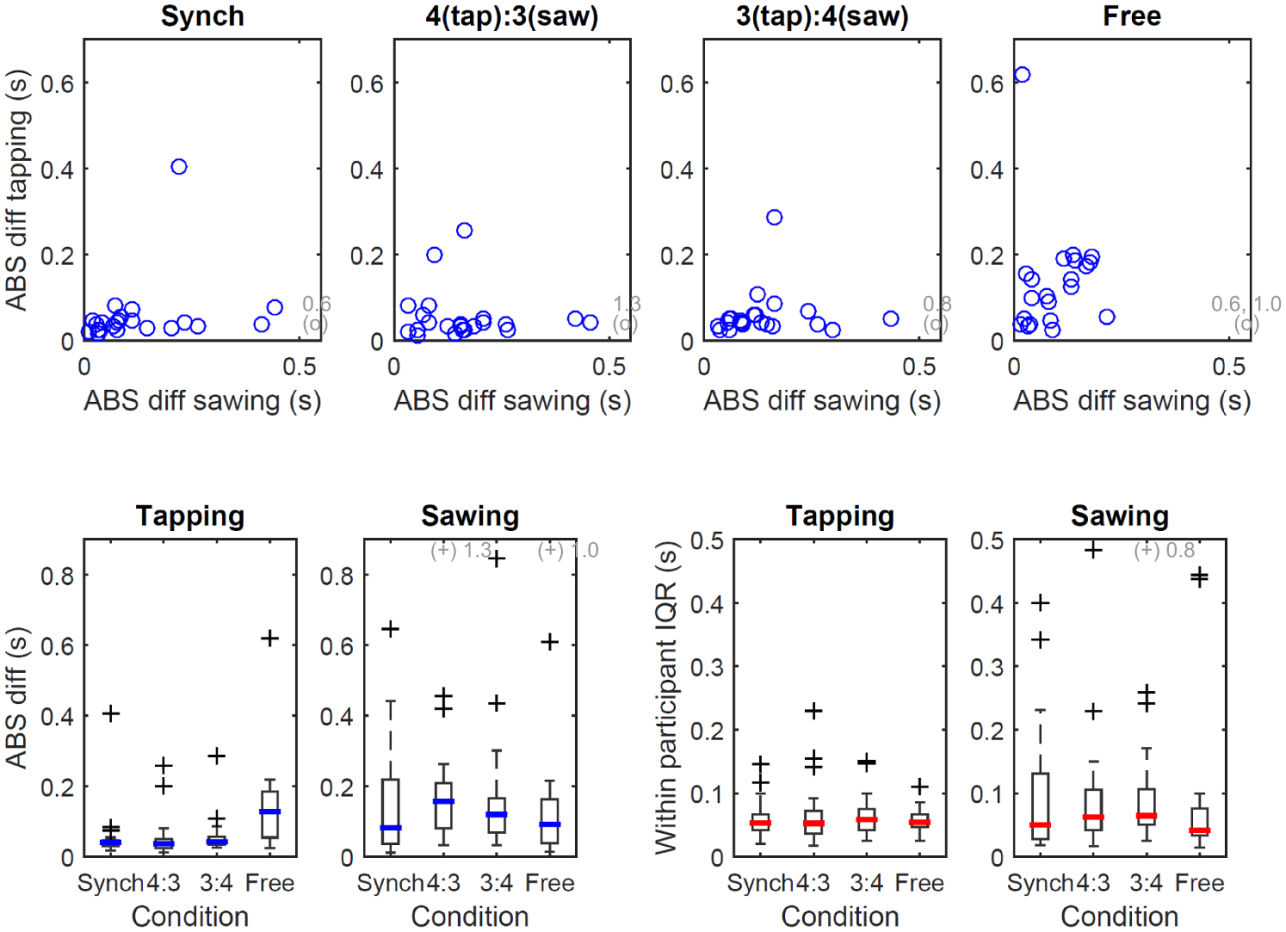
Top: relationship of the median absolute difference between observed and expected cycle duration for the hand engaging in sawing compared to the hand engaging in tapping. Most of the deviation from the expected ratio is due to the difference between expected and observed sawing cycle duration. Bottom: median absolute difference between observed and expected cycle duration (left) and within-participant variation thereof (right) for sawing and tapping. Complementary to the data presented in Fig 3, the deviation from the expected cycle duration is larger for sawing, however the within-participant variation in the deviation is similar for sawing and tapping.

The absolute difference between each cycle duration for each trial and the expected cycle duration is summarised in Fig 5, both for participant-specific median values and within-participant variation (IQR). Most of the deviation from the expected ratio was due to the difference between expected and observed sawing cycle duration rather than a deviation from the expected tapping cycle duration (Fig 5, top). The deviation from the expected cycle duration was larger for sawing, although the within-participant variation in the deviation was similar for sawing and tapping (Fig 5, bottom).

## Discussion

In this study we hypothesised that if simple repetitive actions can be executed at a predictable rate without paying attention to them, then participants would spontaneously produce complex polyrhythms when asked to tap to the varying beats of a metronome whilst sawing. Our results show that this might be the case: without being aware of it, novice participants were able to perform a bimanual polyrhythmic task when tapping four beats to a metronome against three freely executed sawing cycles. The ratio based on the experimental data matched the predicted value, and tapping and sawing were executed at significantly different rates. This substantiates evidence which suggests that untrained novices are able to perform complex polyrhythms in certain conditions. In previous studies, such guidance was applied haptically, sometimes to both hands [25–28]. In our study, we developed the novel experimental paradigm where movement of the hand engaging in sawing was assumed to be performed without the need to pay attention to it or the necessity for conscious control. In this paradigm, we assume movement to emerge predominantly as a feature of the musculo-skeletal system and to a lesser extent follow the natural frequency of an object as described previously [25–28]. The ability to accommodate objects with different inertial properties while performing movements at a consistent rate should be explored in the future to determine how far our findings extrapolate. We expect that there is a point at which cycling at optimal muscle contraction rates will be sacrificed in order to accommodate different loads.

### Spontaneous polyrhythms in context

The observed spontaneously chosen median sawing cycle duration (0.46 s) matched previous findings for sawing [32] as well as the spontaneously chosen tapping cycle duration (0.48 s) observed in this study. This supports our assumption that repetitive hand movements converge on a predictable frequency. Across our bimanual conditions, the sawing cycle duration did not significantly change. Hence, we were able to show that the same effect (spontaneous polyrhythms) as observed for haptically driven movements can arise when one of the actions can be performed without paying attention to it, while the other action, such as tapping, is controlled and systematically altered. These results are similar to those found for haptically guided movements: using methods devised from dynamical systems theory, Treffner and Turvey (1993) describe different polyrhythmic ratios using the ‘Farey Tree’, illustrating how different ratios act as differently strong attractor states towards which people gravitate when performing polyrhythms. In this theoretical framework, a 3:4 ratio is a weaker attractor (Level 3 on the Farey tree) than a 1:3 / 2:3 ratio (Level 2), a 1:2 ratio (Level 1) and a 1:1 / 0:1 ratio (Level 0). By asking participants to oscillate a drumstick at a preferred frequency (‘comfort mode’) while paying attention to tapping to the beat of a metronome with their other hand, the authors showed that ratios which participants were attracted to indeed followed this hierarchy, with many participants shifting away from a 3:4 ratio which was difficult to maintain [28]. In line with the assumption that participants will gravitate towards predictable attractor states when performing polyrhythms, Fig 2 shows a trend towards clustering for the two polyrhythmic tasks around a 1:1 ratio, the predicted 3:4 / 4:3 ratio and a 1:2 ratio.

### Interrelated observations for different ratios

In addition to evidence for spontaneously emerging bimanual independence, we encourage viewing the results in the wider context of the study. We observed that for the ‘synchronous’ condition, participants showed a small bias towards a sawing rate slightly slower than the tapping rate (median [IQR] ratio: 0.83 [0.30], expected: 1), which was significantly different from the expected value and between the two modalities. However, statistically, there was a significant difference between the ratios for the ‘synch’ and ‘4(tap):3(saw)’ conditions, hence the systematic bias did not explain the polyrhythm adopted in the 4:3 condition. We did not have the statistical power to demonstrate a significant difference in movement cycle duration for the 3(tap):4(saw) condition, which showed substantial variation across participants. In a previous study, authors found similarly that for a 3:4 ratio, participants tended to drift towards a 1:1 ratio [37]. When learning to bimanually tap a 3:5 polyrhythm, participants improved mainly by learning to properly tap the slow rhythm component [38]; similarly, tapping the slow rhythm needed most learning in other studies [16,39]. For a 2:3 rhythm, participants performed a lot better when attending to the fast component of the two rhythms [40]. While in our study the slow rhythm component, corresponding to movement of the saw, was assumed to not require conscious control in the 4(tap):3(saw) condition, in the 3(tap):4(saw) condition it would correspond to controlling the finger tap and may have hence required more conscious attention to manage coordination. Within-participant variation also showed a trend to be highest in the 3(tap):4(saw) condition, suggesting that the task was more difficult to execute rhythmically. Hence, our results may suggest that it is easier to maintain a ratio where the consciously controlled movement is faster than the movement executed without the need to pay attention to it. Within-participant variation did not differ substantially between the dual movement conditions, neither between experimental conditions nor between the hand that performed tapping and the hand that performed sawing. Hence, while in the ‘4(tap):3(saw)’ condition participants performed close to the expected ratio, this pattern did not appear to be more stable when within-participant variation is used as an index of stability. The only difference we detected was the within-participant variation observed during single and dual hand movements; in the single hand task, within-participant variation was approximately half that observed during the dual movement tasks. Hence, engaging in the dual movement task increased movement variability of both hands. As Fig 4 shows, the difference in within-participant variation between tapping and sawing was on average close to zero.

### Freely chosen movement cycle durations and performance limits

The freely adopted movement cycle durations for sawing and tapping in this study matched past findings: studies requiring participants to tap their finger at a self-selected rate found an average tapping cycle duration of 0.50 s [41], 0.38 to 0.44 s [42] or 0.52 to 0.63 s across participants from 8 years to 59 years [43, 44], although children as young as 3 years have been reported to spontaneously tap at 0.5 s intervals as reviewed in [10]. Hence, performing hand movements at this rate appears to be a feature that is found across humans at different stages of development. We have previously examined the freely adopted sawing frequency across various participant cohorts and experimental conditions / saws, showing that an average sawing cycle duration of around 0.5 s is consistent [32], matching the 0.46 s cycle duration in the present study. Participants matched the set metronome beat well, with a mean (SD) difference of 0.05 (0.05) s, which highlights the small between-participant variation in the ability to match a given beat. The observed movement cycle durations do not suggest that participants operated near their performance limit, hence limits to feasible cycle durations did not confound the data. For instance, Adults can synchronize tapping to a metronome up to at least 0.29 s intervals [14] or up to at least 0.20 s intervals in a study using visual beat displays [45]. The fastest rate at which humans can tap their finger varies with age; 11-year old children can tap at up to 0.26 s intervals [46], 18 to 25-year old adults can tap at up to approximately 0.16 s intervals [47] and 22 to 34-year olds [41] and 65 to 77-year olds [47] can tap at up to at least 0.20 s intervals. This limit has also been termed the ‘biomechanical limit’ in a review that reported a minimum tapping cycle duration of 0.20 to 0.14 s across various studies [48], with no apparent difference between genders [8].

### Outlook

In the present study, we designed experiments around a 4:3 and 3:4 polyrhythmic structure: these are very difficult to achieve and were hence the benchmark for our hypothesis. Given the variability of data, detecting a statistically significant effect for a relatively small expected effect size is hence a challenge. In the future, examining the coupling between movements which do and do not require attention for different ratios and other tasks would be important in order to explore how our findings extrapolate: based on the assumption that task difficulty is correlated with the ratio of the bimanual movement [18], we would assume that participants can perform ‘easier’ polyrhythms while there may be a limit to the difficulty level at which polyrhythms can be maintained, especially over longer time periods. Tasks which may not include constraints by the environment may be more difficult to perform as part of a polyrhythmic structure: in sawing, the blade is guided by the presence of the wood and the degrees of freedom (DoF) are limited to 2 DoF, respectively vertical and horizontal translation. In comparison, a 6 DoF task may not be possible to control in parallel without paying attention to it. In addition to ratio complexity and task, it will in the future be important to investigate whether polyrhythms are maintained beyond the 15 s task duration tested in the present study, respectively whether findings extrapolate to longer timeframes; it is possible that over longer timeframes, polyrhythms drift towards less complex attractor states [28]. Further, in the present study participants performed sawing with their preferred hand. In a previous study where participants tapped at maximum speed with the preferred hand and matched a metronome with the other hand, performance was much better compared to the reverse scenario [7]. In the future, examining how participants perform when sawing with the non-preferred hand and tapping with the preferred one to test whether performance drops similarly would help shed light on whether control of a bimanual task differs if the preferred or non-preferred hand engages in the assumed automatic task which does not require the main attention.

## Supporting information

S1 Data Data are included in the supporting file. (XLSX)
